# Microbiome First Medicine in Health and Safety

**DOI:** 10.3390/biomedicines9091099

**Published:** 2021-08-27

**Authors:** Rodney R. Dietert

**Affiliations:** Department of Microbiology and Immunology, Cornell University, Ithaca, NY 14853, USA; rrd1@cornell.edu

**Keywords:** chronic disorders, inflammation, human superorganism, holobiont, microbiome, multimorbidity, microimmunosome, polypharmacy, drug safety, sustainable healthcare

## Abstract

Microbiome First Medicine is a suggested 21st century healthcare paradigm that prioritizes the entire human, the human superorganism, beginning with the microbiome. To date, much of medicine has protected and treated patients as if they were a single species. This has resulted in unintended damage to the microbiome and an epidemic of chronic disorders [e.g., noncommunicable diseases and conditions (NCDs)]. Along with NCDs came loss of colonization resistance, increased susceptibility to infectious diseases, and increasing multimorbidity and polypharmacy over the life course. To move toward sustainable healthcare, the human microbiome needs to be front and center. This paper presents microbiome-human physiology from the view of systems biology regulation. It also details the ongoing NCD epidemic including the role of existing drugs and other factors that damage the human microbiome. Examples are provided for two entryway NCDs, asthma and obesity, regarding their extensive network of comorbid NCDs. Finally, the challenges of ensuring safety for the microbiome are detailed. Under Microbiome-First Medicine and considering the importance of keystone bacteria and critical windows of development, changes in even a few microbiota-prioritized medical decisions could make a significant difference in health across the life course.

## 1. Introduction

The human superorganism (also called the human holobiont) is a composite organism composed of the human mammalian body combined with the human resident microbiota (the bacteria, archaea, viruses, and fungi) along with their genes. The various microbiomes inhabit our body across several different locations (e.g., skin, gut, airways, urogenital tract, breast tissue, breast milk). The vast majority of our trillions of microbes are not just friendly to our body but are essential. They provide critical metabolic, physiologic, regulatory and host defense functions (e.g., colonization resistance) as well as vitamins that are needed for life and health [1]. The human microbiota also impacts much of the body’s neurochemical and hormone production, and because of their direct and indirect regulation of neurological function (e.g., via small molecules and epigenetic control), it is sometimes unclear who is really in charge when it comes to the human body [2,3].

Humans are not unique in being mainly microbial in genetic composition. Most higher organisms on earth operate as composites or superorganisms. That is a fundamental nature of much of life on earth (which is primarily a microbial planet) [4]. While the development and function of all of our physiological systems are influenced by the human microbiome, the interaction between the microbiome and the immune system is perhaps the most critical [5]. Part of the critical nature of the microbiome-immune interaction has to do with location. The body sites inhabited by our microbes are the same locations that house a preponderance of our immune cells. Most of these happen to be the mucosal tissues (gut, airways, urogenital tract). The replacement of our immune cells immediately juxtaposed to the microbiota is not happenstance. In fact, the microbes have a lot to do with the recruitment and migration processes that place the immune cells as monitors and sensors for the status of the microbiome [6].

This is particularly evident given that the majority of human immune cells are located in the gut [7], and many of these are separated from the gut microbiota by a one-cell thick epithelial lining and a mucin barrier. The gastrointestinal system is open to the outside world via the mouth and anus. Within the gut, food, drugs, and environmental chemicals are first encountered by the gut microbiota where they are filtered and metabolized for the benefit (or sometimes the detriment) of both the microbiota and our internal tissues and organs.

## 2. The Microimmunosome: A Systems Biology Therapeutic Target

In the gut, the integrity of both the one-cell thick gut epithelial barrier and the mucin lining is critical. They physically separate gut microbes including pathobionts from the underlying immune system, but they also allow bidirectional communication between the two. When something goes wrong with one part of this systems biology unit, (e.g., changes in the microbiota that cause a degradation of the mucin layer), the entire system can be in jeopardy. Microbial dysbiosis can lead to an exposed epithelial barrier. When this barrier is damaged and breached by pathobionts, immune inflammation is initiated and, if uncorrected, local as well as systemic pathology can ensue. In the gut, this is precisely how diseases like metabolic syndrome begin.

Because of the inter-connectivity among the gut microbiota, mucin layer, gut barrier and underlying immune system, it is operationally useful to approach these combined factors as a single systems biology unit. This unit has been termed the microimmunosome [8]. Beyond the gut, the same dynamic exists in the skin, airways, and urogenital tract. These locations (e.g., skin, airways) where microbes meet our immune cells have their own microimmunosomes and should also be approached as well-oiled systems biology units.

When changes occur (e.g., diet, drugs, environmental conditions, lifestyle, stress), it is important that the changes are beneficial for the overall microimmunosome to avoid unintended consequences. For example, a NSAID designed to lower mediators of immune inflammation that has a side effect of producing microbial dysbiosis can easily end up damaging the gut and producing long term increases in both the risk of infections and immune inflammation. Short term gains in modulation of inflammation can boomerang to longer term systemic problems.

In medicine and healthcare, it is the microimmunosome that is the ultimate target. Changing one element of the systems biology unit will inevitably impact the other components of the unit. The physician needs to be aware of this relationship and manage prevention and therapies accordingly.

## 3. The Immune System and Superorganism Integrity

The discovery of the microbiome and its role in molding our immune development and function has led to a 21st century rethinking of what the immune system is really designed to do. No longer is the immune system viewed as put into existence to detect and kill all microbes. Instead, the immune system grows up with the developing human microbiome, prunes the microbiome, surveils the microbiome, directly reflects microbiome development and status, and responds accordingly when the microbiome is damaged and dysbiotic. Damage to the microbiome can occur via high fat or sugary diets, toxic food additives (e.g., emulsifiers), toxic environmental chemicals (e.g., pesticides) and even microbe-damaging drugs (e.g., proton pump inhibitors [8]). Of course, incompleteness of or damage to the human microbiome predictably leads to immune-inflicted damage to our bodies [9,10].

There is a new 21st century dogma that views humans as mainly microbial (genetically) and fundamentally a superorganism (where our body is a composite of thousands of different species). This dogma changes the very definition of what it means to be healthy. Being healthy within our 21st century understanding means existing in a specific microbe-rich state. In contrast, illness is the state of being out-of-balance and biologically incomplete. We are ill when we become something akin to a damaged coral reef. Illness is the state of being poorly microbially balanced (often lacking certain commensal microbes) and lacking the colonization resistance necessary to control against pathobiont attack [11].

As for the immune system, we need to upgrade our thinking to adjust to immune biology and medical support in this era of the superorganism. The same approaches that we pursued in the 20th century no longer hold. It is time to discard 20th century dogmas about microbes and the immune system and to embrace the microimmunosome as a focal point when it comes to preventing and managing diseases [12].

**Figure 1:**
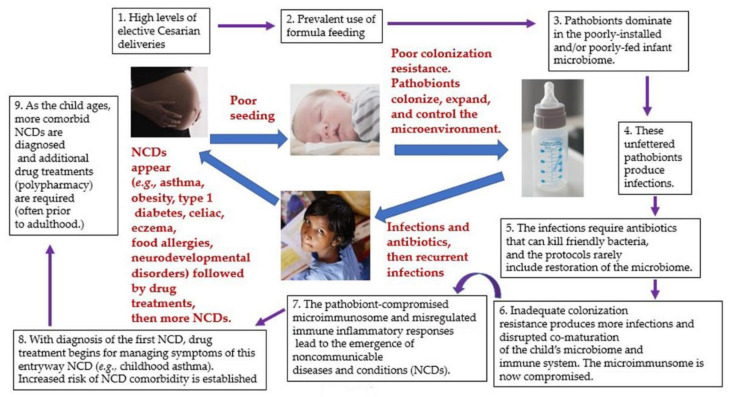
Lack of focus on the microbiome promotes a pathobiont-dominated microbiome, a damaged microimmunosome, misregulated inflammation, and the likelihood of multimorbid NCDs over the life-course.

It should be noted that many 20th century-established, medically-approved practices are an ongoing problem. They are a problem because they prevent patients from acquiring a healthy microbiome and/or deplete and damage their existing microbiome [13]. Dietert [13] considered the initial sequence of disease-promoting events. Figure 1 illustrates how our current single-species-oriented medical management can lead the child toward a healthcare intensive, illness-filled life.

This disturbing sequence of events has given us: (1) ever-increasing numbers of chronic diseases across the life course, (2) polypharmacy, (3) increased risk of life-threatening infections, (4) reduced human capacities, (5) reduced quality of life, (6) increased need for caregivers, and (7) unsustainable medical costs. There is a better way.

## 4. Introducing Microbiome First Medicine

This paper introduces the concept of Microbiome First Medicine as a strategy to reverse the NCD epidemic and move us toward sustainable healthcare. Because the human microbiome carries the vast majority of our genes [14], fundamental human biology dictates that the microbiome should be front and center in any preventative or therapeutic medical plan. It is counterproductive to focus on protection of health and treatment of chronic diseases involving a patient’s internal organs/physiological systems, while at the same time failing to address microbiome dysbiosis in the human superorganism.

## 5. NCDs: Human Illness and Death

NCDs can be life changing and life threatening. They are the leading cause of global death (71% of all deaths as reported by the World Health Organization) [15]. But beyond the eventual cause of death for most individuals, these diseases and conditions both restrict function and activities and can require specific medications that may be prescribed for life. One example of this is statins prescribed to lower cholesterol [16].

Many diseases such as Alzheimer’s disease, Parkinson’s disease, dementia, multiple sclerosis, cystic fibrosis, fibromyalgia, autism, stroke, and cancer can require caregivers. This can be a significant burden for families in terms of financial obligations and stress. In fact, caring for patients with NCDs can affect the health and well-being of the caregivers [17,18].

The epidemic of NCDs over the past several decades has been seemingly tolerated within healthcare as we became an all too passive observer of NCD proliferation. Progress in medical specialties involving single organs/physiological systems and drugs medically coded for disease-specific use can mask a bigger picture. New NCD distinctions have been teased out, and new medications were developed for each new diagnosis. Unfortunately, with NCDs as a growth industry, core causes, and dysfunctional commonalities among NCDs became largely lost in the weeds. Yet, potential cures reside in those commonalities among NCDs (e.g., microbiome dysbiosis and misregulated inflammation). That is why, if healthcare is to be sustainable, it needs to focus on the patient from the outside-in beginning with restoration of the microbiome.

## 6. The Immune System as a Manager of Superorganism Integrity

The primary function of the immune system is to identify and survey the components of the human superorganism and to ensure that the body’s tissues and organs maintain integrity, structure, and function. Tissues and organs have to function within certain limits and commensal microbes need to be tolerated while pathobionts are held in check. It is useful to think of the different human microbiomes as another organ or tissue that the immune system has to survey and manage.

The concept of maintaining tissue integrity as the core function of the immune system was described by Dembic [19] and is simple enough. Of course, our tissues are different, and have diverse functions, while specialized resident cells of the immune system must grapple with that diversity. However, when tissue integrity includes the human microbiome and trillions of essential microbes doing their job, ensuring tissue integrity becomes even more challenging.

## 7. NCDs as Mainly Immune-Inflicted Diseases

The majority of global deaths worldwide are caused by chronic diseases also known as noncommunicable diseases and conditions (NCDs). These are the prevalent diseases of asthma, heart disease, obesity/diabetes/metabolic syndrome, cancer, neurological degenerative conditions, autoimmune diseases, allergic diseases and inflammatory conditions (arthritis, psoriasis, frailty, etc.). It was recently recognized that the term noncommunicable is less useful than previously thought. At least some of the NCDs appear to be transferable (i.e., communicable) to others via specific pathobionts when the recipient’s microbiome is compromised and receptive [reviewed in 12]. Therefore, noncommunicable appears to be contextual and not absolute.

NCDs are inherently immune-inflicted in nature. The immune system literally destroys parts of the body in response to organismal composite imbalances. The inappropriate immune response is the body’s attempt to “defend and repair” the human superorganism in the face of systems biology disruptions connected to microbiome dysbiosis. To exist and persist, these chronic diseases, while in many different tissues, have a common feature. They require ongoing, unresolving, immune-driven inflammation. The misregulated immune system does not like what is presented and attacks with auto-destruction as a result [20].

Note that many medical/pharmacological treatments for chronic disease such as asthma, heart disease, inflammatory bowel disease, etc. attempt to modulate inflammatory mediators and their impact on tissues and organs. Few if any treatments actually go to the heart of the cause: misregulated inflammation resulting from human superorganism dysbiosis.

## 8. Two High-Impact Examples of NCDs

To highlight the risk of receiving an NCD diagnosis within our current healthcare system, two common NCDs are considered: asthma and obesity. These are two highly prevalent diseases/conditions with a predictable path of both treatments and future comorbid diseases.

### 8.1. Asthma

Asthma is a chronic respiratory disease of both allergic and non-allergic origins. While it can emerge at any time in life, the first episode of asthma is often preceded by one of several generic respiratory virus infections [21]. This disease is among the earliest group of NCDs to be seen during childhood. Asthma is part of the allergic triad of diseases, which includes allergic rhinitis (hayfever), and atopic dermatitis. More recently, food allergies have been added to that allergic complex and have become another important childhood-onset disease.

Table 1 shows examples of 36 comorbid NCDs where asthmatics are at a higher risk of being diagnosed with these NCDs vs. the general population [22,23,24,25,26,27,28,29,30,31,32,33,34,35,36,37,38,39,40,41,42,43,44,45,46,47,48,49,50,51,52,53,54,55,56,57,58]. The 36 comorbidities of asthma range well beyond the allergic conditions. Remarkably, they include autoimmune, metabolic, cardiovascular, neurobehavioral, neurodegenerative, endocrine, reproductive, systemic conditions (e.g., mast cell activation), cancer (e.g., lung) and even what are termed end-stage diseases (e.g., frailty). The asthmatic child has a life course that is literally bookended by NCDs unless and until we approach medicine and healthcare differently.

Asthma is one of the diseases where symptoms are controlled often by multiple prescribed medications. But actual cures have evaded us. This disease has several different endotypes usually differing in the mix of the immune system components that infiltrate the lungs and mediate inflammation and pathology in the airways. In severe forms, asthma is a truly debilitating disease. A recent finding is that asthma can be caused by and exacerbated by a pathobiont (a potentially infectious agent). In this case it is the gram-positive bacterium, *Staphylococcus aureus* (Staph A). The bacterium can exist in a hospital-associated, multi-drug resistant form called Methicillin-resistant *Staphylococcus aureus* (MRSA). When Staph A is a predominant colonizer in the nasal passages of infants, those infants are at a higher risk of childhood asthma [59]. But when the microbiome is healthy, other bacteria can block Staph A from taking up residence via colonization resistance [60,61]. Managing the infant microbiome to optimize colonization resistance against Staph A is one way to reduce the risk of childhood asthma. But this requires, Microbiome First Medicine.

While asthma is a significant enough life burden by itself when left uncured, it is the high risk of other comorbid NCDs that makes a pediatric asthma diagnosis a major life-threatening event. Asthma and other allergic diseases should be considered as an entryway ticket to what constitutes a web of additional childhood- and adult-onset comorbid NCDs.

### 8.2. Obesity

Obesity is one of the major conditions that gives rise to a myriad of comorbid, inflammation driven, chronic diseases particularly as aging progresses [62,63]. Table 2 illustrates examples of 43 comorbid NCDs for obesity [64,65,66,67,68,69,70,71,72,73,74,75,76,77,78,79,80,81,82,83,84,85,86,87,88,89,90,91,92,93,94,95]. The obese population carries one of the highest risks for multiple future NCDs of any disease-burdened cohort. As a component of metabolic syndrome, obesity can arise at any time in life. However, it is one of the NCDs that is frequent in childhood and, as a result, can be a disease burden and comorbid disease risk for most of the life course [96].

Obesity is a significant contributor to end stage diseases such as chronic kidney disease [97] and frailty (a pro-inflammatory end stage condition associated with muscle loss) [98]. Among the immune changes that help to spread obesity, macrophage populations undergo significant changes that can lead toward the various manifestations of metabolic syndrome [99]. One of the theories behind inflammation spread with obesity is that adipocyte-derived extracellular vesicles may disrupt redox signaling and facilitate the spread of inflammation to the cardiovascular system [100].

Similar to the case with asthma, application of Microbiome First Medicine in obesity could interrupt our current life course march toward increasing numbers of comorbid diseases. Because of the web of connected diseases, if we do nothing different in medicine and continue to manage NCD symptoms rather than restoring integrity to the microbiome and immune system, the NCD epidemic will only continue if not get worse.

## 9. Marching to Multimorbidity and Polypharmacy through a Web of NCDs

The outcomes shown in Table 1 and Table 2 can only be described as unacceptable results stemming from the medical mismanagement of two epidemic NCDs, asthma and obesity. It is possible that most asthmatics and obese patients and their healthcare providers are not fully aware of this reality. Nor are they necessarily aware that asthma and obesity are themselves linked together as bidirectionally-shared comorbidities.

A look beyond asthma and obesity to gastrointestinal (e.g., inflammatory bowel), neurological (e.g., multiple sclerosis), metabolic (e.g., type 2 diabetes), reproductive (PCOS), cardiovascular (e.g., atherosclerosis), and dermal (e.g., psoriasis) NCDs would lead one to conclude that most NCDs have a large number of comorbidities, and that current medical management of these diseases has produced similar outcomes as illustrated for asthma and obesity. For example, when inflammatory bowel disease and psoriasis were examined for comorbid diseases more than a decade ago, serious comorbid NCDs were identified [101]. A second finding was that many NCDs share depression, sleep disorders, and atherosclerosis as comorbidities [101]. More recent evaluations of inflammatory bowel disease and psoriasis found that both diseases have double digit NCD comorbidities [102,103,104].

The problem with the NCD epidemic is not just the fact that 71 percent of people globally die of NCDs [15]. It is what happens along the way between the cradle and the grave. The progression towards this type of death inevitably runs through multimorbidity (carrying two or more NCDs) and polypharmacy. The prevalence of multimorbidity among different populations in the U.S. was determined through the National Health and Nutrition Examination Survey (NHANES) and could be compared across several different years [105]. In the 2013–2014 survey, the prevalence of two or more NCDs in all adults (age 20 and higher) was 59.6%. For the young adult 20–44 age group, more than one third had multimorbidity (37.5%), in the 45–64 age group it was 70.6%, and for the age 65 and older group, it was, remarkably, 91.8%. Clearly, many U.S. adults are aging over decades carrying a significant NCD burden. These diseases are not being cured. Rather, the symptoms are being managed amid what is a growing disease burden.

Polypharmacy is not without its own problems. Beside the fact that it is not sustainable at a global level in treating multi-comorbid-burdened patients, evidence suggests that polypharmacy is associated with reduced cognitive function [106]. Hence, reduced quality of life and increased caregiver needs are the rewards for additional prescriptions.

## 10. Drug Safety for the Human Superorganism

The problem with the NCD epidemic and polypharmacy is not simply the number of prescription drugs that an individual accumulates with aging, it is that the existing drugs were also produced and vetted with only the human mammal in mind. The patient was the human mammal, and the safety of drugs medically coded for each NCD did not extend to the human microbiome [107].

Table 3 shows examples of commonly used drugs that interact with and in many cases damage the human microbiome [108,109,110,111,112,113,114,115,116,117,118,119,120,121,122,123]. In a recent investigation, approximately half of all commonly used drugs affected the microbiome [110]. For some drugs, metabolism by the microbiota is required for the drug’s active form to be produced. The human microbiome varies such that if a physician does not know the patient microbial composition, the appropriateness of the drug and/or the drug dose is also unknown.

In some cases, such as with the cardiac drug, digoxin, the lack of knowledge about the patient’s microbiome could result in the administered drug being problematic and potentially lethal [126]. The levels of a single gut bacterium, *Eggerthella lenta*, determine the pharmacokinetics of digoxin (see Table 3). This bacterium can metabolically inactivate digoxin and affect the internally-delivered dose of the drug [126]. Hence, the internal dose of the active drug can differ significantly from the physician-delivered dose. This is where personalized, Microbiome First Medicine becomes important. The physician needs to know the patient’s microbial metabolic activity for digoxin in order to be able to prescribe an effective, non-lethal dose.

Another pattern of drug-microbiome interactions is when the drug selectively kills part of the microbiome. Selective killing of commensal bacteria can damage colonization resistance and enable other microbes to grow in an unrestricted manner. Loss of colonization resistance significantly affects the risk of pathobiont-driven infections (e.g., MRSA, *Clostridioides difficile*). This can happen with common drugs such as non-steroidal anti-inflammatory drugs (NSAIDs) [127] and proton-pump inhibitors [128].

There are two take home messages from this information. (1) For patient safety and drug efficacy, drugs should be prescribed under the personalized medicine rubric with the patient’s microbiome in mind. (2) All new drug candidates should be demonstrated to be safe for the human microbiome.

## 11. Microbe Management: Keystone Species and Cooperative Metabolic Communities

At first glance the idea of practicing microbiome first medicine (optimizing the human body working from the microbiome inward) might seem daunting. After all, the human microbiome is complex with trillions of microbes taking up residence in and on various parts of our body.

But there is evidence that benefit can come from medically simplifying the microbiome. A starting point is to divide the specific microbiome (i.e., gut, skin, airways, urogenital tract) into two basic groups: (1) the keystone species and (2) communities of microbes, or metabolic modules, that together create a useful metabolic environment in a specific body site. The keystone species are indispensable microbial species that each provide a unique function. Prolonged damage to a keystone species usually results in disease. In contrast, the communities of microbes (site-specific metabolic modules) provide important functions based on group interactions. Specific microbes within a metabolic module are often interchangeable. Within these modules, significant health risks occur when there are changes in group function rather than changes in a single microbial species. The metabolic modules are identified using approaches such as metabolomics [129] and metabologenomics [130]. Analysis of a patient’s keystone species and microbial metabolic module status can guide physicians on making adjustments to both the microbiota and supportive microbial food sources (prebiotics/diet).

Keystone species in the microbiome are specific, often unique microbes that provide a vital function. They are largely irreplaceable, and the function provided is usually a tipping point between health and disease. If the physician, healthcare provider, and patient know nothing else about the microbiome, they should know the status of keystone species. While there are several keystone species among the body’s microbiomes, two gut keystone bacteria deserve special mention: *Bifidobacterium longum* ssp *infantis* and *Akkermansia mucuniphila*. *B. infantis* is both the premier metabolizer of the food component of human breast milk [human milk oligosacharrides (HMOs)], and it is a pivotal orchestrator of infant immune maturation [131,132].

*A. muciniphila* is the premier regulator of the gut’s mucin layer, which protects the one-cell thick gut barrier and helps to guard against improper immune activation and hyperinflammation [133]. To prevent childhood and adult NCDs, the pediatrician needs to ensure that these keystone functions are in place in the infant to balance the immune system, protect the gut barrier, and reduce the risk of immediate infections followed by future NCDs.

Cooperative metabolic communities provide site-specific metabolic modules that optimize a variety of functions in specific regions of the body site. In the case of these communities of microbes, some of the parts (e.g., bacterial species) may be redundant or interchangeable. But a combination of microbes with a useful mix of genes provides functions ranging from colonization resistance against pathobionts, to neuroactive peptide synthesis, to short chain fatty acid (SCFA) production, to bile acid metabolism. Such community microbial function is significant for the patient’s overall physiology and well-being (i.e., brain, immune, gut, liver, and other tissue functions). Not surprisingly for cooperative metabolic communities, it does come down to “location, location, location” and microbial communities can create healthful ecological niches within the human gut. Here is where metabolically-driven readouts aid the physician/healthcare provider. Because the microbiome, its genes, and metabolic status are fully adjustable, the physician and patient are both empowered and poised to make useful, impactful changes. But they can only do this if they know to look to the microbiome first and foremost, and know what adjustments would bring the patient into functional balance.

## 12. Blocking Pathobionts to Protect against NCDs

Blocking pathobionts using a multi-level, front-line defense known as colonization resistance is beneficial across the entire spectrum of disease challenges. This is one of the most effective yet under-utilized tools within current preventative medicine. Under Microbiome First Medicine, this tool would become routine across medical specialties with the immediate effect of reducing the need for antibiotics.

Effective colonization resistance goes well beyond just stopping infectious diseases from happening. As discussed by Dietert and Dietert [12], the dogma stating that there is a “boundary” between NCDs and communicable (infectious) diseases is proving to be more like an outdated 20th century construct than a 21st century reality. Many NCDs are actually communicable within the context of the dysbiotic microbiome [12,134]. It is now clear that microbiome dysbiosis not only allows pathobionts to breach barriers and infect, but that many of those same pathobionts have the capacity to cause quite specific NCDs when the microimmunosome has been compromised.

As introduced in Dietert [135] and expanded here, at least three examples exist of pathobiont-produced NCDs are known. The gram-positive bacterium *Staphylococcus aureus* (Staph A) (carried in the nose, skin, and the urogenital tract) can directly promote asthma using multiple direct and indirect changes to the immune system [59,136]. Adherent-invasive *Escherichia coli* is another human pathobiont that can produce the inflammatory bowel disease “NCD” under the circumstances of microbiome dysbiosis [137].

Beyond these two examples, Rath et al. [138] present a list of what have been termed pathogenic functions among various human pathobionts. Many of these pathobionts over-produce metabolic products that result in NCDs. A prime example of this is the production of the microbial metabolite trimethylamine (TMA), which can lead to cardiovascular disease (particularly atherosclerosis), type 2 diabetes, and renal disease [138]. TMA is at the heart of the conversion of macrophages into “foam cells,” which sit at the center of atherosclerotic plaques. TMA is significantly associated with *Enterobacteriaceae* bacteria with a subset of *Clostridiales* contributing community production of the metabolite [139,140].

## 13. Critical Windows for Programming Health vs. Disease

Early prenatal and postnatal development is known to be a period of vulnerability during which significant programming occurs for childhood and adult health vs. disease [141,142]. This idea originated with what became known as the Barker hypothesis when Robert Barker (a British MD) demonstrated that prenatal development could program for later-life cardiovascular disease [143]. Soon, it was clear that the programming window extended to significant periods of postnatal development and included programming for most NCDs [144]. For this reason. attention to the infant microbiome status and immune co-maturation process is critical for preventative healthcare.

One problem is that *Clostridiales* and *Enterobacteriaceae* bacteria along with *Staphylococcus* are among the original colonizers in premature babies and immediately after birth in full term newborns [145]. It is critical that these founding bacteria not persist, and that they be replaced by the *Bifidobacterium* group and in particular, *B. longum* spp *infantis. B. infantis* must be introduced into the infant gut for two reasons. First, the prior colonizers contain potential pathobionts and TMA producers. *B. infantis* lowers the pH providing colonization resistance against the pathobionts and preventing enteric inflammation from occurring [146,147].

This is a critical developmental window for both the gut microbiome and the immune system. A priority for medical care should be to ensure that the newborn-infant is colonized with *B. infantis*, and that these bacteria are fed so as to become predominant in the gut. Fortunately, of all bacteria *B. infantis* is a major metabolizer of human milk oligosaccharides (HMOs), a major component of breast milk [147]. These complex sugars cannot be digested by our cells and are only there to feed the gut microbes. Hence, the baby needs HMO metabolism by *B. infantis* to receive nourishment from breastfeeding, and the baby needs *B. infantis* to protect against pathobionts and their dangerous metabolites. The added benefit to the baby by having pediatricians focus on this transition is that it would automatically reduce the potential exposure to TMA and, as a result, reduce the risk of later life atherosclerosis for that baby.

This is a prime example of how Microbiome First Medicine can produce immediate benefits in reducing health risks in the infant with an added longer-term benefit of preventing diseases that would emerge during adulthood. It is a completely different approach from the current trend of treating presenting symptoms and chasing after an ever-increasing number of comorbid diseases as the patient ages.

## 14. Probiotics, Prebiotics and Targeted Rebiosis: Clinical and Preclinical Examples

Significant efforts are underway both to prevent and to treat communicable diseases and NCDs by optimizing the microbiome. Installation and/or growth promotion of selected microbiota have been used to: (1) avoid the programming of disease and (2) provide critical microbial copartners necessary for the effective treatment of NCDs.

Table 4 illustrates examples from human clinical trials [131,148,149,150,151,152,153,154,155,156,157,158,159,160,161,162,163,164,165,166,167,168,169,170,171,172,173,174,175,176,177,178,179,180,181,182,183]. Not surprisingly, the best outcomes occur when there is a clear understanding of the specific microbes, genes, metabolic changes, and installation conditions that are needed to support microbiome-driven physiological benefits. But the range of benefits in human cohorts from direct management of the microbiome means that this approach can no longer be dismissed as too experimental. The era has passed in which the microbiome could simply be ignored in the practice of medicine.

Preclinical research on targeted microbiome alterations is shown in Table 5 [184,185,186,187,188,189,190,191,192,193,194,195]. The range of these studies presages where Microbiome First Medicine is headed. In particular, the power of being able to provide microbiome-initiated solutions to systems biology health problems is evident. Conversely, attempts at systems biology fixes while the microbiome remains in dysbiosis are questionable and are unlikely to produce permanent cures.

## 15. Revisiting the Microimmunosome: Bidirectional Approaches for Homeostasis

An important feature of the microimmunosome is bidirectional communication between the immune system and the microbiome. Bidirectional signaling operates regardless of the microbiome body location (e.g., gut, skin, airways, urogenital tract). For this reason, once systems biology units like the microimmunosome are locked into an inflammation-promoting state with likely tissue pathology, the entire systems unit needs to be rebalanced.

There are many examples in the literature where changes in the immune system can affect the human microbiome [196]. Knowing the mediators of the bidirectional immune-microbiota cross talk can aid preventative strategies and therapeutic approaches.

The primary antibody of mucosal tissues (IgA) is a case in point when it comes to bidirectional communication and influence within the microbiome. Through reciprocal regulation, microbiota can affect, in part, IgA production by exerting control over regulatory T cells and T helper follicular cells [197,198]. In return, IgA applies selective pressures on gut microbiota. The IgA antibody promotes symbiotic bacteria cooperation within the microbiome [199,200,201] and selectively targets colitis-inducing gut bacteria, thereby, reducing inflammation [202,203]. Microbiota-IgA bidirectional regulation acts as a type of feedback loop [204,205].

Specialized immune cells can also affect microbiota composition. For example, subpopulations of innate lymphoid cells (ILC-1, ILC-2, and ILC-3) respond to and control different pathobionts across the human microbiomes. In the gut, ILC- 1 cells control of *Salmonella typhimurium* in the gut, in the stomach, ILC-2 control *Helicobacter pylori*. In the lungs, the ILC-3 subpopulation controls *Streptococcus pneumoniae* [206]. While ILC-2 is a responder to *Staphylococcus aureus* in the skin [207].

With bidirectional communication across the microimmunosome, it is possible to approach NCD treatments starting at either end of the systems biology unit and monitoring for effects across the microimmunosome. Treatment of metabolic syndrome is an example. Evidence suggests that both ends of the microimmunosome (the microbiota and immune cells) are useful targets. In mice, Wang et al. [208] administered a mix of fourteen composite probiotics to rebalance the microbiome. This microbiome-based treatment corrected insulin secretion, blood glucose metabolism, barrier function, and eventually immune dysfunction. Macrophage polarization was shifted from M1 polarization to M2 simply by changing the microbiome. Other investigators have had success starting at the opposite end of the microimmunosome. They treated metabolic syndrome by infusing M2 polarized macrophages into obese mice where M1 macrophages were predominant in adipose tissue. By simply rebalancing polarization of tissue macrophages, beneficial effects were seen in a reverse direction across the microimmunosome. Metabolic parameters were improved when the only adjustments had involved macrophages [209,210,211]. Recent information suggests that bile acid metabolism is a critical link that binds together gut microbiota, metabolic status, and tissue macrophage polarization to determine normal physiology vs. metabolic syndrome [212,213,214].

Given the bidirectional effects between the microbiome and physiological systems, it is useful to question the cause-effect relationships between physiological and/or organ toxicities and microbiome damage and/or altered metabolism. A classic example where the microbiome’s role was only recently discovered is for the heavy metal lead (Pb). Lead produces abnormal function in the neurological and immune systems with extreme vulnerability during early life [215,216]. Pb is also a timely toxicant for consideration following the disastrous mass population exposure via drinking water in Flint, Michigan [217,218].

More than a half a century of research on lead-induced immunotoxicity defined many aspects of exposure-outcomes and health risks across the life course [219,220,221,222,223]. But recent findings suggest that the microimmunosome may provide a better vantage point for accessing health risks and treatment options. In a review, Liu et al. [224] reported that Pb exposure affects all elements comprising the microimmunosome. It produces dysbiosis of gut microbiota, impairs the gut barrier and increases permeability. Pb exposure also alters bile acid metabolism and short chain fatty acid production. The researchers pointed out that Pb-induced inflammation and immune dysregulation could result directly from Pb exposure or via the altered gut microbiome and loss of barrier function. They concluded that: (1) the microbiota are likely to be the first “victims” of Pb exposure and (2) probiotics appear to facilitate lead excretion and provide therapeutic benefits following Pb-induced toxicity. A second research group showed that specific Pb-intolerant bacteria protected against internal Pb exposure by facilitating Pb excretion and reducing blood lead levels [225]. Taken together, these recent findings support the utility of the microbiome as a sentinel of problematic exposures and a biomarker of underlying health risks.

## 16. Challenges for Microbiome First Medicine

There are numerous challenges ahead for implementing a Microbiome First Medicine approach as the gold standard for human health protection and the treatment of chronic disorders. It seems clear that we have more to learn about the microbiome in the future than we know now. Almost each month new research shows us that the human microbiome is affecting and often controlling more and more aspects of our body’s functions than were previously known. Gaps in knowledge exist for the human microbiome even as they exist for each of our physiological systems. But it is clear that we know enough and have already seen enough microbiome-based benefits to readjust our medical focus.

While some health care providers are already focused on the health and protection of the human microbiome, they are in the minority. Additionally, the support for their still-novel practices is dwarfed by the status quo as is reflected in pharmaceutical advertising. The formula needs to be inverted such that knowledge of and focus on the human microbiome is the medical and public health default among practitioners. It starts with an assemblage of stakeholders who fully understand that we are more than simply one species, a human mammal, and that we must be whole across the lifespan to thrive.

Those stakeholders need to include: (1) One Health educators and administrators in medical and veterinary schools, (2) leaders in public health institutions, foundations and granting agencies, (3) clinicians, and other health care service providers, (4) academic, government and industry researchers, and finally, the most important group, (5) the patients. Many microbiome-oriented groups already exist. But their information has mainly been available to those who seek it out. As stakeholders we must ensure that those who practice medicine and healthcare have the tools necessary to assess and manage the microbes of their patients.

Above all, those leaders who have advocated for personalized and precision medicine, who recognize that we cannot afford to continue growing the world’s number one killer, NCDs, and who state that we need sustainable healthcare, must also embrace and help us protect our various microbiomes. If one examines the public health choices that occurred during the ongoing pandemic, one of the “side effects” has been human microbiome degradation, rather than restoration [226,227]. The outcomes of human microbiome degradation are well known [228] and are discussed among this and other papers in this special issue. Human microbiome degradation is not a path toward sustainable healthcare.

## 17. Conclusions

Chronic disorders such as NCDs are extensively interlinked by dysbiosis, comorbidity, and misregulated inflammation. Additionally, communicable and noncommunicable diseases and conditions are not as separate as was once thought. There are microbes that can produce NCDs under permissive conditions when microbiome dysbiosis exists and colonization resistance is compromised.

Because the human microbiome is located at the routes of exposure for many drugs, food and environmental chemicals as well as occupying sites that are the portal of entry of infectious agents, the microbiome should be the starting point for patient management. Everything else literally flows downstream (into the tissues) from these gatekeeping microbiota. They almost exclusively determine the spectrum of drug, food and environmental chemical metabolites that reach our body’s internal tissues and organs, and they determine all-important biologically relevant internal doses. They are the keepers of our exposome across the lifespan. It is important that: (1) the human microbiome is protected from drugs, medical procedures, and environmental factors that result in dysbiosis of the microbiome and disease [229] and (2) support for microbiome be used to optimize the effectiveness of medical treatments [230]. For these reasons, management of a patient’s microbiome is key to both disease prevention and the delivery of safe, effective levels of therapeutics to the internal body

Microbiome First Medicine starts with three basic components:

1. Knowledge of a patient’s prior and current microbiome status
2. Probiotic installation in the patient to:
  A. Facilitate key developmental events during infant development (e.g., microbiota needed to digest human milk and reduce NCD-promoting inflammation)
  B. Provide enhanced colonization resistance and reduced risk of infections
  C. Correct a potential physiological imbalance (e.g., hormonal, immunological, neurological, G.I., hepatic, renal, or reproductive related)
  D. Reduce the risk of a comorbid NCDs
  E. Aid the effectiveness of a medication
  F. Reduce side effects of a medication
  G. Improve multi-system functionality in circumstances of neurological disorders (e.g., brain-gut)
  H. Reduce the risk of cytokine storm in the event of certain infections
3. Dietary and/or prebiotic alterations to support the microbiome overall and any adjustments made to the microbiota

## Figures and Tables

**Table 1:** Comorbidities of Asthma.

Disease, Condition, and/or Extrapulmonary Symptom	Reference(s)
Allergic rhinitis	[22]
Atopic dermatitis	[23]
Food allergies	[24,25]
Chronic obstructive pulmonary disease (COPD)	[26]
Chronic sinusitis	[27,28]
Obesity	[29]
Depression	[26]
Anxiety	[26]
Mood swings	[30]
Attention Deficit Disorder	[31]
Fatigue	[30]
Gastro-esophageal reflux disease (GERD)	[32]
Osteoarthritis	[33]
Vocal cord dysfunction	[34]
Stomach ulcers	[35]
Nasal polyps	[36]
Frailty	[37]
Hypertension	[38]
Skeletal muscle wasting/Sarcopenia	[39]
Mast cell activation syndrome	[40]
Hormone imbalances/disorders	[41,42]
Type 2 diabetes	[43]
Obstructive sleep apnea	[44]
Insomnia/sleep disorders	[45,46]
Ischemic heart disease	[47]
Hypoxia	[48]
Stroke	[49]
Acute myocardial infarction	[50]
Posttraumatic Stress Disorder (PTSD)	[51]
Myasthenia gravis	[52]
Lung cancer	[53]
Allergic bronchopulmonary aspergillosis (ABPA)	[54]
Type 1 diabetes ^1^	[55]
Metastasized breast cancer ^2^	[56]
Eosinophilic granulomatosis with polyangiitis (vasculitis) ^3^	[57]
Coronary heart disease ^4^	[58]

^1^ Some subtypes of asthma in children. ^2^ The metastasized form of breast cancer is more likely in asthmatics because of inflammation-promoted metastasis. ^3^ Associated with severe asthma. ^4^ More frequent in females with adult-onset of asthma.

**Table 2:** Examples of Comorbidities of Obesity.

Disease or Condition	Reference
Type 2 diabetes	[64]
Hypertension	[65]
Coronary artery disease	[66]
Atherosclerosis	[67]
Polycystic ovary syndrome (PCOS)	[68]
Urinary stress incontinence	[69]
Osteoarthritis	[70]
Dyslipidemia	[71]
Obstructive sleep apnea	[72]
Sleep fragmentation	[73]
Nonalcoholic fatty liver disease (NAFLD)	[74]
Nonalcoholic hepatic steatosis (NAHD)	[75]
Asthma	[76]
Rheumatoid arthritis	[77]
Crohn’s disease (in women)	[78]
Hearing loss	[79]
Deep vein thrombosis	[80]
Infertility	[81]
Frailty	[82]
Alzheimer’s disease	[83]
Gout	[84]
Hypothyroidism	[85]
Dementia	[86]
Multiple sclerosis	[87]
Schizophrenia (cortical thickness reduction)	[88]
Chronic obstructive pulmonary disease (COPD)	[89]
Depression	[90]
Anxiety	[91]
Psoriasis	[92]
Attention Deficit Disorder	[93]
Chronic kidney disease	[94]
Leukemia	[95]
Uterine cancer	[95]
Gallbladder cancer	[95]
Thyroid cancer	[95]
Cancer of the cervix	[95]
Hepatic cancer	[95]
Ovarian cancer	[95]
Postmenopausal breast cancer	[95]
Colon cancer	[95]
Kidney cancer	[95]
Pancreatic cancer	[95]
Rectal cancer	[95]

**Table 3:** Examples of Drug Interactions with the Microbiome.

Drug/Drug Category	Damages and/or Interaction(s)	Reference(s)
Digoxin (Cardiovascular)	Internal drug dose affected by gut bacterium, *Eggerthella lenta*	[108,109,110]
Non-steroidal anti-inflammatory drugs (NSAIDs)	Produces NSAID-specific enteropathy, damage to specific microbiota that protect against gastric enteropathy; Probiotics may help functionality	[111,112,113]
Proton Pump Inhibitors	Damages approximately 20% of the gut microbiota; increases the risk of enteric infections	[112,114,115]
SSRI Antidepressants	SSRIs act like antibiotics completely restructuring the gut microbiome causing loss of some needed species and overgrowth of others	[112,116,117]
Oral steroids	Can cause overgrowth of obesogenic, methogenic bacteria	[112]
Metformin (Type 2 diabetes)	Increased growth of *E. coli* species with increased risk from pathobionts	[112,118]
Laxatives (with polyethylene glycol)	Reduced diversity of the gut microbiome	[119]
Beta Blockers	Increase in pathobiont for dental caries, *Streptococcus mutans*	[118]
H1 inhibitor antihistamines	Increase in *Clostridium bolteae* a pathobiont associated with both gut and neurotoxic metabolites	[118,120]
Platelet aggregation inhibitors (aspirin)	Increases in several Streptoccocus and Clostridial pathobionts and reduction in a major GABA producer (*Bifidobacterium adolescentis*)	[118,121]
Opioids (Pain management)	The gut microbiome becomes dysbiotic and can lock in the dependency.	[122,123]
Cancer therapeutics	The gut microbiome can be altered by cancer therapeutics and this can subsequently affect immune anti-tumor effectiveness. Also the cancer drug, cyclophosphamide, has a better prognosis for lung cancer patients when the patients carry the bacterium, *Enterococcus hirae*	[124,125]

**Table 4:** Examples of Recent Use of Probiotics/Rebiosis in Disease Prevention and Therapy.

Probiotic/Rebiosis Strategy [Reference(s) in brackets]	Disease Prevention/ Therapy	Specific Effects
*Bifidobacterium longum* ssp *infantis* EVC001 [131]	Protection against pathobiont-induced enteric inflammation in the infant	Protection against enteric inflammation and reduction in proinflammatory cytokine levels promoted by *Clostridiaceae* and *Enterobacteriaceae*
Multiple different probiotics [148]	Protection against antibiotic-associateddiarrhea (AAD)	Meta-analysis of 36 studies including 9312 participants showing a 38% reduction in disease incidence.
Multiple probiotic regimes [149]	Protection against post-operative adverse outcomes from colon cancer surgery	Meta analysis of six studies with 457 subjects. Probiotic administration improved gut barrier and colon function, reduced inflammatory markers, and increased gut microbiome diversity with increased colonization resistance post- surgery
Probiotic cocktail (*L. plantarum* MH-301, *B. animalis* subsp. *Lactis* LPL-RH, *L. rhamnosus* LGG-18, and *L. acidophilus)* [150]	Protection against oral mucositis (OM) following nasopharyngeal cancer chemoradio-therapy	In a clinical trial of 77 patients, probiotic supplementation resulted in a significantly lower prevalence of OM and a reduced severity grade.
Multiple probiotic regimes [151]	Protection against side effects of chemo and radiation cancer therapies	Meta-analysis of 20 studies, 17 studies produced positive results in reducing side effects while three studies found no significant differences.
Multiple probiotic regimes [152]	Protection against osteoporosis in post-menopausal women	Meta analysis of five randomized controlled trials with 497 participants. Probiotic supplements significantly increased bone mineral density in the lumbar spine.
Multi-strain probiotic [153]	Protection against gastrointestinal symptoms among elite athletes	Reduced gastrointestinal symptoms and distress following intense training sessions competition
Reconstituted milk powder containing the probiotic, *Lactobacillus paracasei* SD1 [154]	Protection against new dental caries and Reduction in existing dental carieslesions	Beneficial preventative and therapeutic oral health effects in preschool children
Multiple probiotic regimes [155]	Protection against hospital acquired *Clostridioides difficile* infection	Meta-analysis of 19 prevention studies with 6261 subjects. The infection rate was decreased by greater than 50% from controls.
Multiple probiotic regimes [156]	Protection against *Clostridioides difficile*-associated diarrhea in children and adults	Meta analysis with complete case analysis of 31 trials with 8672 participants. The risk of C. difficile infection was reduced by 60%.
Oral Fecal Microbiota Transplant (FMT) [157]	Protection against recurrent *Clostridioides difficile* infection and Long term treatment of *C. difficile* infected patients	Meta analysis of efficacy of oral FMT capsules. 15 studies with 753 patients had an efficacy rate of 82.1%
Multiple probiotic regimes [158]	Protection against Necrotizing enterocolitis (NEC) in premature infants	Meta-analysis of 51 studies. *Lactobacillus acidophilus* LB was the most successful of probiotics used in reducing the risk of NEC
Different probiotic combinations across the trials [159]	Protection against complications following colorectal cancer surgery	Meta analysis of 15 trials, Improved mucosal protection/function and microbial diversity following antibiotics
Probiotic fermented dairy products [160]	Protection against respiratory infections	Meta analysis of 22 clinical trials with 10, 290 participants. Overall infection rate was decreased by 18–21% across children (18%), adults (19%), and elderly (21%)
Microbiota transfer therapy [161,162,163,164]	Therapy for autism spectrum disorders	Significant improvements in gastrointestinal symptoms, autism functionality and gut microbiome metabolism and diversity
*Bifidobacterium breve* BR03 and B632 strains [165]	Therapy for obesity/insulin resistance	Improved metabolic parameters; decreased weight; improved insulin sensitivity
*Bifidobacterium longum* APC1472 [166]	Therapy for obesity	Improved fasting blood glucose levels
*Hafnia alvei probiotic strain HA4597^®^* [167]	Therapy for obesity	Significantly improved weight loss; feeling of fullness; reduction in hip circumference; Fasting glycemia
*Lactobacillus rhamnosus* GG (LGG) [168]	Therapy for coronary artery disease (CAD)	Reduced metabolic endotoxemia and mega inflammation
Multiple probiotic regimes [169]	Therapy for type 2 diabetes	Meta-analysis of 26 studies with 1947 participants. Probiotics significantly reduced the glycemic index in type 2 diabetics.
*Lactobacillus paracasei* HII01 [170]	Therapy for type 2 diabetes	Decreased plasma blood glucose levels with reduced inflammatory markers and restored gut microbiota profile and function
Multi-strain probiotics [171]	Therapy for type 1 diabetes (children)	Improved glycemic control
Multiple different synbiotics, Meta-analysis [172]	Therapy for ulcerative colitis	Beneficial reduction in inflammation; Reduced inflammatory cytokines andC-reactive protein levels; elevated levels of anti-inflammatory cytokines
Fecal Microbiota Transplantation [173]	Therapy for active ulcerative colitis	Following three treatments, increased levels of gut *Faecalibacterium prausnitzii* bacterium and a significantly reduced Mayo Clinic score for UC.
Mix of *Lactobacillus acidophilus* (TYCA06), *Bifidobacterium longum* subspecies *infantis* (BLI-02), and *B. bifidum* (VDD088) [174]	Therapy for chronic kidney disease	Attenuation of renal function deterioration and reduction in inflammation
*Mixture of Lactobacilli and Bifidobacteria* strains [175]	Therapy for atopic dermatitis	Reduced clinical severity; reduced intestinal inflammation
*Lactobacillus rhamnosus* CGMCC 1.3724 [176,177]	Therapy for food allergy (peanuts)	Sustained tolerance when combined with peanut oral immunotherapy
*Bifidobacterium bifidum* TMC3115 [178]	Therapy for food allergy (Cow’s milk)	Probiotic treatment of infants ameliorated the allergy with lower allergy scores, elevated anti-inflammatory responses, lower serum IgE and higher IgG2
*Bifidobacterium animalis* Subsp., *Lactis* BB12 and *Enterococcus faecium* L3 [179]	Therapy for allergic rhinitis (AR)	Prevention of signs and of required use of medications among children. With prophylactic probiotic treatment begun three months before allergy season, the signs and symptoms of AR were significantly reduced as was use of drugs, including oral antihistamines and local corticosteroids.
*Bifidobacterium* mixture (*B. longum* BB536, *B. infantis* M-63, *B. breve* (M-16V) [180]	Therapy for allergic rhinitis and intermittent asthma	Improved symptoms and quality of life
Multi-strain Synbiotic [181]	Therapy for viral infections in asthmatic children	Reduced number of viral infections vs. placebo group
*Lactobacillus rhamnosus* SP1 [182]	Prevention of and therapy for dental caries	Consumption of probiotic-laden milk reduced the prevalence of dental caries in children
*Lacticaseibacillus rhamnosus* GG and *Saccharomyces cerevisiae boulardii* [183]	Used for restoration of the gut microbiome following antibiotic treatment	Increases in major bacteria groups and short chain fatty acid production postantibiotics

**Table 5:** Examples of recent preclinical trials and research with probiotics.

Probiotic/Rebiosis Strategy (Species) [Reference(s)]	Disease Prevention/Therapy	Specific Effects
*Faecalibacterium prausnitzii* (mouse) [184]	Allergic asthma	Immune cell and cytokine normalization, reduced airway pathology, increase short chain fatty acid production; enhance gut microbiome diversity
*Bifidobacterium longum* ssp *infantis* (mouse) [185]	Allergic asthma	Probiotic supplementation reduced ovalbumin-specific IgE antibodies, reduced infiltration by inflammatory cells, and shifted cytokine levels from Th2 to Th1.
Mixed probiotics (mouse) [186]	Allergic asthma	Immune-based alleviation of allergic asthma, reduced inflammation with restoration of the gut microbiome
*Bifidobacterium longum* ssp *infantis* (mouse) [187]	Nasal allergy	Probiotic promotion of IL-10 producing dendritic cells that suppressed the nasal allergy.
*Lactobacillus brevis* FZU0713-fermented *Laminaria japonica* (rat) [188]	Obesity	Significantly inhibited obesity and improved serum and hepatic biochemical parameters in High fat diet-fed rats. Changes in both gut microbiota composition and short chain fatty acid production
Miso-derived *Zygosaccharomyces sapae* strain I-6 -yeast (mouse) [189]	Colitis	Significant reduction in inflammation by production of IL-10 from dendritic cells
Consortium of probiotics (mouse) [190]	Atherosclerosis	Blocked atherogenic processes via immunomodulation
*Lactobacillus casei* adjunct therapy (mouse) [191]	Malaria	Blocked parasitemia
Yeast-based engineered, self- tunable probiotics (mouse) [192]	Inflammatory bowel disease	Reduced inflammation, intestinal fibrosis and dysbiosis
*Lactobacillus paracasei* and *Lactobacillus plantarum* (mouse) [193]	Chronic kidney disease	Improved kidney function with reduction in kidney injury and fibrotic-related proteins and restoration of gut microbiota
*Bifidobacterium bifidum* FSDJN7O5 and *Bifidobacterium breve* FHNFQ23M3 (mouse) [194]	Diarrhea caused by enterotoxigenic *Escherichia coli*	Alleviation of symptoms with restoration of gut function and physiology
*Bifidobacterium bifidum* G9-1 (BBG9-1) (rat) [195]	Maternal separation used as a model of the colonic mucosal problems as seen with irritated bowel; M1-macrophage driven inflammation	Probiotic administration protected against M1 macrophage-driven adverse effects. There were reduced numbers of M1 macrophages with increased CD-80 positive cells; reduced inflammatory cytokine production; the probiotic administration protected and stabilized the colonic mucosa
